# Between guidelines and clinical trials: evidence-based advice on the pharmacological management of non-specific chronic low back pain

**DOI:** 10.1186/s12891-023-06537-0

**Published:** 2023-05-30

**Authors:** Filippo Migliorini, Raju Vaishya, Gaetano Pappalardo, Marco Schneider, Andreas Bell, Nicola Maffulli

**Affiliations:** 1 Department of Orthopaedic, Trauma, and Reconstructive Surgery, RWTH University Hospital of Aachen, 52064 Aachen, Germany; 2 Department of Orthopaedic and Trauma Surgery, Academic Hospital of Bolzano (SABES-ASDAA), Bolzano, 39100 Italy; 3 Department of Orthopedics, Indraprastha Apollo Hospitals Institutes of Orthopaedics, New Delhi, India; 4 Department of Spine Surgery, 14482 Oberlinhaus, Potsdam, Germany; 5 Department of Medicine and Dentistry, University of Witten/Herdecke, 58455 Witten, Germany; 6 Department of Arthroscopy and Joint Replacement, MVZ Praxisklinik Orthopädie Aachen, RWTH University Hospital Aachen, 52074 Aachen, Germany; 7 Department of Orthopedics, Eifelklinik St. Brigida, Simmerath, Germany; 8 Department of Medicine, Surgery and Dentistry, University of Salerno, Baronissi, 84081 Italy; 9 Barts and the London School of Medicine and Dentistry, Queen Mary University of London, Mile End Hospital, London, E1 4DG England; 10 School of Pharmacy and Bioengineering, Stoke on Trent, Keele University Faculty of Medicine, Keele, England

**Keywords:** Spine, Low back pain, Chronic, Pharmacological therapy

## Abstract

The pharmacological management of nonspecific chronic low back pain (NCLBP) aims to restore patients’ daily activities and improve their quality of life. The management of NCLBP is not well codified and extremely heterogeneous, and residual symptoms are common. Pharmacological management should be considered as co-adjuvant to non-pharmacological therapy, and should be guided by the symptoms reported by the patients. Depending on the individual severity of NCLPB, pharmacological management may range from nonopioid to opioid analgesics. It is important to identify patients with generalized sensory hypersensitivity, who may benefit from dedicated therapy. This article provides an evidence-based overview of the principles of pharmacological management of NCLPB.

## Introduction

Nonspecific chronic low back pain (NCLBP) is the single most common cause of pain and disability in industrialised countries and a common cause for consultation in primary care. The world population of adults aged 60 years or older is expected to double by 2050, and NCLBP might become a major public health burden worldwide [1]. The prevalence of NCLBP in adults aged 60 years or older is approximately 36.1%, with an annual incidence of 15 to 20% in the United States [2, 3]. The prevalence of NCLPB in women is double compared to men [4, 5]. Up to 95% of low back pain is not attributable to a formal anatomically based aetiology and is therefore defined as nonspecific [6–8]. Low back pain is considered “chronic” when symptoms last more than three months [9–12]. Though chronic LBP may develop after a definite injury, in many patients no precipitating event can be identified [13–15]. Pharmacological management should be considered as co-adjuvant to non-pharmacological therapy if symptoms still do not promote relevant improvement in the quality of life of the patients, considering the risk of adverse events [9–12, 16–34]. Assessment of patient general health, including blood tests to evaluate kidney and liver function, is recommended before pharmacological therapy is started, and repeated systematically to monitor the possible occurrence of adverse events.

The management of NCLBP is not well codified and is heterogeneous, and residual symptoms, such as stiffness, muscle spasms, and axial back pain, are common [35, 36]. Internationally accepted recommendations and guidelines are still missing. This review aims to provide an evidence-based overview of the principles of pharmacological management of NCLPB.

Management of NCLBP.

Pharmacological management aims to restore patients’ daily activities and improve their quality of life. No magic bullet exists for NCLBP; pharmacological interventions to manage pain and disability are available with acceptable short-term results [37]. However, their long-term efficacy is unpredictable. Tolerance, desensitisation, and addiction are a concern in patients managed pharmacologically. This is often hard to accept for clinicians and patients and provides fertile soil to quacks, faith healers, and gurus to promote miraculous non-evidence-based solutions. Clinicians have to choose from drugs with very modest effects and variable risk profiles [38]. Hence the widespread recommendations to use pharmacological options as a last resort [39]; the benefits are just not there to justify the routine of prolonged use of any drug in NCLBP [38, 40, 41]: this is the major challenge for clinicians. The pharmacological management of patients with NCLBP should be individualised according to the patient’s symptoms, but at present little evidence is available. Recommendations from guidelines are heterogeneous (Table 1) with also within-country differences.

**Table 1:** Overview of international guidelines (CCS: corticosteroids; NSAIDs: non-steroidal anti-inflammatory drugs; SSRI: Serotonin-Noradrenalin-Reuptake-Inhibitors; TCAs: tricyclic antidepressants)

Guidelines	NSAID (selective)	NSAID (non selective)	Paracetamol	Opioids (weak)	Opioids (strong)	CCS	Antibiotics	Duloxetine	Gabapentinoids	SSRIs	TCAs	Myorelaxans
Canada [30]	*favour*	*favour*	*favour*	*inconclusive*	*inconclusive*	*against*	*against*	*against*	*against*	*against*	*favour*	*favour*
USA [42]	*favour*	*favour*	*against*	*favour*	*favour*	*inconclusive*	*against*	*inconclusive*	*-*	*-*	*-*	*against*
USA, Canada, Chile, Switzerland [43]	*inconclusive*	*inconclusive*	*against*	*inconclusive*	*inconclusive*	*against*	*-*	*against*	*against*	*-*	*inconclusive*	*against*
North America [44]	*inconclusive*	*favour*	*against*	*inconclusive*	*inconclusive*	*against*	*against*	*against*	*inconclusive*	*-*	*against*	*against*
USA [45]	*inconclusive*	*inconclusive*	*inconclusive*	*against*	*against*	*against*	*-*	*favour*	*inconclusive*	*-*	*favour*	*inconclusive*
ICSI/USA	*inconclusive*	*inconclusive*	*inconclusive*	*inconclusive*	*inconclusive*	*inconclusive*	*inconclusive*	*-*	*-*	*inconclusive*	*inconclusive*	*-*
Denmark [12]	*inconclusive*	*inconclusive*	*inconclusive*	*inconclusive*	*inconclusive*	*inconclusive*	*inconclusive*	*-*	*-*	*inconclusive*	*inconclusive*	*-*
UK [46]	*favour*	*favour*	*against*	*favour*	*inconclusive*	*inconclusive*	*-*	*-*	*against*	*against*	*against*	*against*
France [47]	*favour*	*favour*	*favour*	*favour*	*favour*	*inconclusive*	*against*	*-*	*against*	*against*	*against*	*against*
Germany [9]	*favour*	*favour*	*against*	*favour*	*favour*	*-*	*-*	*-*	*-*	*-*	*-*	*-*
Belgium [48]	*favour*	*favour*	*against*	*favour*	*inconclusive*	*inconclusive*	*against*	*-*	*against*	*against*	*against*	*against*

A medical history of the characteristics of pain is required, as previous positive experiences or undesired adverse events may influence treatment prescription. The visual analogue scale (VAS) or numeric rating system (NRS) should be used to monitor pain intensity and therapy efficacy. Non-pharmacological approaches, including manipulation, education and psychological strategies, and structured physical activity programs, are recommended as first-line therapy for chronic low back pain [9–12, 16–31, 34, 49–52]. Physical therapy aims to improve function and prevent disability, and should be considered first-line therapy in patients with NCLBP [53, 54]. Despite the several heterogeneous physiotherapy regimes advocated, a consensus on the best modality has not yet been reached. Kinesiotherapy, specifically global postural re-education using the Souchard [55] or Mezieres [56] methods, massages and manipulation, trunk muscle activation, and strengthening and mobilization of soft tissues are recommended [57]. Moreover, other non-physical therapies have been introduced for the management of NCLBP, including dry needling, manual therapy, acupuncture, and McKenzie exercises [42, 48, 58]. Given the neuropathic mechanisms which largely contribute to NCLBP and the oxidative stress which might cause nerve damage typical of neuropathic pain [59], antioxidants administration has been proposed as an adjuvant in the multimodal management of NCLBP. Ozone therapy has been also advocated in NCLPB [52]. A highly oxidizing oxygen-ozone mixture causes a controlled “micro oxidation” that produces a modulation of the cellular antioxidant system and the inflammation system could increase tissue diffusion, immunomodulation, and analgesia, and could reduce oedema [60–62]. Computer tomography-guided oxygen ozone infiltration combined with oral administration of alpha-lipoic acid (ALA) with palmitoylethanolamide (PEA) and myrrh can be also considered as an adjuvant for the management of NCLBP [63, 64]. Similarly, also alpha-lipoic acid (ALA), superoxide dismutase (SOD), and PEA have been advocated to improve neuroprotection and reduce symptoms [65–68]. Previous evidence showed that the oral combination of ALA and SOD might improve function and reduce the use of analgesics in NCLPB [69, 70]. The action of PEA, especially if combined with complementary and alternative medicine therapies, may also represent a valid additional non-pharmacological alternative for NSLCBP, especially if resistant to conventional therapies [71, 72].

## Symptomatic therapy for NCLPB

Some guidelines advocated the stepwise administration of paracetamol (acetaminophen), non-steroidal anti-inflammatory drugs (NSAIDs), and opiates [20, 22, 24]. Considerable uncertainty exists about the clinical efficacy of paracetamol for NCLBP, with limited evidence and low-level recommendations. Current guidelines worldwide are contradictory: most guidelines recommend the use of paracetamol [10, 25, 27–30, 73], some guidelines advise against it [9, 11, 12, 16, 17, 19, 23, 34, 50]. Compared to a placebo, the administration of paracetamol did not promote any improvement in pain or disability. Following these considerations, the Agency for Clinical Innovation (ACI) approve the use of paracetamol in NCLBP but advises that it might not be effective [74, 75]. The evidence supporting the use of paracetamol is limited [13, 75, 76]. However, paracetamol can be considered an adjuvant in combination with other drugs, especially opiates [77–80].

The use of NSAIDs in NCLBP has been supported by high-quality evidence [13, 80–89] and is advocated by several guidelines [11, 19, 20, 22, 24, 26–30, 34, 73, 90, 91]. To minimize adverse effects, the lowest effective dose for the shortest possible period is recommended [89]. NSAIDs target in a more or less reversible fashion the binding site of the two isoforms of cyclooxygenase (COX): COX-1, which is constitutively produced and ubiquitous under physiological conditions, and COX-2, which is inducible and present mostly in the active inflammatory phase. Two types of NSAIDs are available: selective and non-selective for COX-2. Non-selective NSAIDs, while reducing inflammation and platelet aggregation (especially aspirin), increase the risk of gastrointestinal ulcers and bleeding [92, 93]. Selective COX-2 inhibitors have fewer gastrointestinal effects [94, 95], but they promote thrombosis and substantially increase the risk of a heart attack. Consequently, selective NSAIDs are generally contraindicated in patients with a higher risk of cardiovascular and renal diseases, and non-selective NSAIDs in patients with a higher risk of gastrointestinal ailments.

Current evidence demonstrated that opioids promote clinically relevant benefits in pain and disability in the short term (with most placebo-controlled RCTs ≤ 6 weeks in duration) [77, 83, 96–110]; however, evidence on long-term opioids administration is lacking, and they are unlikely to exert clinically important effects in patients with NCLBP, even at dangerously high doses [38]. Their administration should be cautiously considered, given their high risk of abuse, addiction, tolerance, and desensitisation [111–113]. Other major risks associated with the use of opioids include cardiovascular events, endocrinologic ailments, increased risk of road accidents, hormonal changes and life-threatening respiratory depression [114–117]. Nausea, confusion, constipation, and hyperalgesia are minor adverse effects, but are common among patients who use opioids [118, 119]. Current guidelines recommend the use of weak opioids in NCLBP for the shortest period in chronic LBP if other analgesics are contraindicated, not tolerated, or ineffective [9, 11, 19, 20, 27–29, 50]. [120–123]. Opioid therapy should be attempted when the benefits for pain and function are expected to outweigh the risks [124–127]. If opioids are used, they should be combined with nonpharmacological therapy and nonopioid pharmacological therapy [128–130]. At the time of opioid therapy setting, clinicians should establish realistic treatment goals with their patients [131–133]. Immediate-release opioids at the lowest effective dosage should be preferred instead of extended-release/long-acting opioids [109, 134]. If pain and function do not improve as desired, opioid therapy should be discontinued [134–136]. In general, dose increases that do not provide sustained improvement should be reversed [137, 138]. Weak opioids (e.g. tramadol, tilidine/naloxone) should be attempted at first [137, 139]. Strong opioids (e.g. oxymorphone, buprenorphine) should only be considered as a last resort within a multimodal therapy framework and in cooperation with specialists in pain therapy [109, 111, 140, 141].

The use of corticosteroid administration in NCLBP is not supported by the current evidence [26, 142], with no established administration protocol. Long-term corticosteroid administration should be avoided, as they induce hyperglycaemia, osteoporosis, immunosuppression, sexual dysfunction, hypertension, depression, and other hormonal dysfunctions [143]. We advise against antibiotics administration for NCLBP, which demonstrated no benefit in NCLBP and elevated risk of adverse events, especially subsequent microbiological resistance [27, 144, 145]. The use of antibiotics was based on the hypothesis that NCLBP may be induced by a low-grade infection [145], but this has not been verified.

## Central sensitisation

NCLBP affects both physical and mental health. The latter is impacted by long-term anatomical, biochemical, and functional alterations to the central nervous system that affects the way pain is modulated and perceived, as central sensitization is often present [146, 147]. Central sensitization is defined as “an amplification of neural signalling within the central nervous system that elicits pain hypersensitivity” [148], “increased responsiveness of nociceptive neurons in the central nervous system to their normal or subthreshold afferent input” [149], or “an augmentation of responsiveness of central neurons to input from unimodal and polymodal receptors” [150]. Despite this difference, all these definitions agreed on the neurophysiological mechanism of increased response to stimuli in central nervous system neurons responsible for the lower pain thresholds, larger receptive fields, and abnormal pain in response to external stimuli [151–155]. In addition, patients with central sensitization from NCLBP may also present chronic pain in multiple body areas [156, 157]. Sensitization in the central nervous system may perpetuate pain even in the absence of anatomical damage [158–161]. Despite improved knowledge of the processes leading to central sensitization, it is still difficult to diagnose [162, 163]. Methods to classify pain aetiology and predominance (e.g. central sensitization, neuropathic, or nociceptive) have been developed [164, 165]; however, no guideline or validated diagnostic algorithm has been yet developed. A targeted therapy addressed to this generalized sensory hypersensitivity may be considered in isolation or combined with the first-line symptomatic therapy [160, 164, 166]. There is little evidence that duloxetine, an analgesic inhibiting serotonin and norepinephrine reuptake, may promote improvement in pain and disability in NCLBP [37, 40, 167–171]. Given its central pain pathway inhibition [172], duloxetine should be administered in patients with a greater central sensitization component as second-line co-therapy [40], especially in those patients with multiple and chronic painful sites. Flupirtine is an aminopyridine central acting non-opioid analgesic with no anti-inflammatory properties [107, 173]. Flupirtine demonstrated efficacy in the management of NCLBP [107] and also in other acute and subacute musculoskeletal pain ailments [174, 175]. However, given its high risk of hepatotoxicity, potential addiction, and unclear benefit, flupirtine administration for NCLBP should be cautiously monitored. Guidelines from Germany advise against the use of flupirtine in NCLBP [19]. As fatigue is very common following flupirtine administration, the ability to operate a motor vehicle is impaired [176]. The present evidence should benefit from further high-quality investigation to attest to the efficacy and safety profile of flupirtine for NCLBP. The current evidence on Gabapentinoids (gabapentin and pregabalin) administration for NCLBP is limited and demonstrates the considerable risk of adverse effects, including drowsiness, somnolence, dizziness, ataxia, fatigue, respiratory depression, and if combined with opioids, they might promote cardiac insufficiency [83, 177, 178] without proved benefit [79, 83, 84, 177–179], with high costs, and addiction risks [180] and is not supported by current guidelines [11, 19, 20, 26, 27]. Gabapentinoids administration for NCLBP merits caution and further high-quality investigations are required. Overall, the evidence is not yet sufficient to recommend the use of anticonvulsive drugs in the treatment of LBP [40, 181]. Indeed, topiramate in the management of NCLBP did not induce improvement [182, 183]. The use of topiramate has limited evidence [184], and is not recommended by several guidelines [11, 19, 20, 26, 27]. Most guidelines advised against the use of antidepressants in NCLBP [11, 19, 20, 26, 27]. Selective Serotonin-Noradrenalin-Reuptake-Inhibitors (SSNRIs) should not be used on a regular basis, and only if relevant psychiatric comorbidities (severe depression, anxiety disorder) justify their use [19, 90]. Tricyclic antidepressants (TCAs) interfere with serotonin reuptake and binding receptors, interacting with α2-adrenoreceptors (noradrenergic system). TCAs differentially regulate opioid receptors with a preferential agonist activity, which may contribute to their therapeutic and/or side effects [185]. Other important effects of TCAs are the binding to N-methyl-D-aspartate (NMDA) and amino-3-hydroxy-5-methyl-4-isoxazole propionic acid (AMPA) glutamate receptors, the inhibition of peripheral adenosine uptake, and the block of sodium channels. TCAs are not effective as a single therapy in the management of NCLPB [186–191], and should be administered only as co-therapy in patients with neurological pain [22], as they did not score better than placebo in the management of NCLBP [186, 192].

## Myorelaxants

Although muscle tension and spasm may be a primary or secondary cause for LBP, the administration of central myorelaxants in NCLBP is controversial. While prescribed to relieve pain arising from muscle spasms, muscle relaxants might offer short-term pain relief, but have no effects on muscle spasm itself, and their effectiveness is uncertain [32, 33, 193]. Previous evidence demonstrated that non-benzodiazepine myorelaxants might promote minimal improvement in NCLBP at approximately two weeks in isolation or as co-medication [40, 181, 194]. Recommendations for central myorelaxants are variable: some guidelines recommend them for the management of NCLBP as second line therapy in exacerbations [10, 26, 28, 30], and others discourage their administration [9, 19, 20, 24, 27, 29, 31, 34]. However, given their multiple collateral effects and interactions, the risk for addiction and tolerance, along with the limited evidence, central myorelaxants should be only indicated for short-term acute exacerbation in NSCLPB under closed monitoring. Future studies should investigate validated methods to identify and quantify the muscular component of NCLBP, to establish proper candidates for the administration of central myorelaxants.

## Conclusion

The management of NCLBP is not well codified and is extremely heterogeneous, and residual symptoms are common. A non-pharmacological approach is a first-line therapy for NCLBP.

Pharmacological management should be considered as co-adjuvant to non-pharmacological therapy and should be guided by the symptoms of the patients. Depending on the individual diagnostic picture, pharmacological management may range from nonopioid to opioid analgesics. Current guidelines are heterogeneous, with a variable range of indications and contraindications, with contrasting recommendations. For a proper management of NCLBP, further high-quality RCTs and shared guidelines are required.

## Data Availability

Not applicable.

## Abbreviations

NCLBP: nonspecific chronic low back pain
VAS: visual analogue scale
NRS: numeric rating system
NSAIDs: non-steroidal anti-inflammatory drugs
COX: cyclooxygenase
SSNRIs: selective serotonin-noradrenalin-reuptake-inhibitors
TCAs: tricyclic antidepressants
NMDA: N-methyl-D-aspartate
AMPA: amino-3-hydroxy-5-methyl-4-isoxazole propionic acid
